# Symmetry Analysis of Gait between Left and Right Limb Using Cross-Fuzzy Entropy

**DOI:** 10.1155/2016/1737953

**Published:** 2016-02-24

**Authors:** Yi Xia, Qiang Ye, Qingwei Gao, Yixiang Lu, Dexiang Zhang

**Affiliations:** ^1^School of Electrical Engineering and Automation, Anhui University, Hefei 230601, China; ^2^Information Technology Research Centre, Nanjing Sport Institute, Nanjing 210014, China

## Abstract

The purpose of this paper is the investigation of gait symmetry problem by using cross-fuzzy entropy (C-FuzzyEn), which is a recently proposed cross entropy that has many merits as compared to the frequently used cross sample entropy (C-SampleEn). First, we used several simulation signals to test its performance regarding the relative consistency and dependence on data length. Second, the gait time series of the left and right stride interval were used to calculate the C-FuzzyEn values for gait symmetry analysis. Besides the statistical analysis, we also realized a support vector machine (SVM) classifier to perform the classification of normal and abnormal gaits. The gait dataset consists of 15 patients with Parkinson's disease (PD) and 16 control (CO) subjects. The results show that the C-FuzzyEn values of the PD patients' gait are significantly higher than that of the CO subjects with a *p* value of less than 10^−5^, and the best classification performance evaluated by a leave-one-out (LOO) cross-validation method is an accuracy of 96.77%. Such encouraging results imply that the C-FuzzyEn-based gait symmetry measure appears as a suitable tool for analyzing abnormal gaits.

## 1. Introduction

Human gait is a complex process. The locomotor system incorporates input from the cerebellum, the motor cortex, and the basal ganglia, as well as feedback from visual, vestibular, and proprioceptive sensors [1]. Under healthy conditions, this multilevel control system produces a periodic and complementary movement of legs, which can be further subdivided into eight sequential subphases [2]. Following this delicate control strategy, a considerable degree of symmetry or similarity exists on the moving cadence and the stride length between left and right limb [3]. However, factors such as aging [4], peripheral neuropathy [5], and neurodegenerative disorders [6] could undermine such control mechanism in normal gait and lead to disturbance of gait phases and inconsistent stride length and disrupt rhythm. As a result, increased stride-to-stride variability and asymmetry often happened in abnormal gait [7, 8]. Hence, gait analysis was an important component during the clinical diagnosis or therapy assessment for those gait-related diseases [9].

During the past decades, with the rapid development of sensor technology and the emergence of corresponding signal processing methods, a lot of research efforts have been devoted to providing a quantitative and long-term gait evaluation methodology [2, 8, 10, 11]. Our main interest in this study is the quantitative assessment of gait symmetry, which has been addressed in several different studies [8, 12–15]. Among these studies, one frequently used clinical measure of symmetry [12, 15, 16] is ASI = 100*∗*(*T*_*R*_ − *T*_*L*_)/(0.5*∗*(*T*_*R*_ + *T*_*L*_)), where *T*_*R*_ and *T*_*L*_ are the values of feature *T* that is extracted from a time series for right and left limb, respectively. The degree of symmetry can also be quantified by the Pearson correlation coefficient between two time series, and an example of such studies was presented by Su et al. [13]. In addition to the above two methods, gait symmetry was also investigated by other methods. Sant'Anna and Wickström [14] proposed a symbol-based gait symmetry measure. Liao et al. [8] introduced multiresolution entropy analysis into the evaluation of gait symmetry.

Though most methods have achieved a certain success, relatively few studies have tried to apply cross entropy to the analysis of gait symmetry. Cross entropy is a kind of complexity measure that is generalized from entropy. By definition, the cross entropy measures the synchrony of two time series as the studies in [17–19] have done, but it can be also used for measuring the degree of dissimilarity of two concurrent, nonstationary biological signals [20–22]. In this study, the application of cross entropy to the evaluation of gait symmetry is inspired by the following observation: a symmetric gait must be similar, and a certain degree of dissimilarity must exist in an asymmetric gait. Therefore, by way of measuring gait similarity, the gait symmetry can also be measured.

From the literature, the first cross entropy, that is, cross-approximate entropy (C-ApEn), was proposed by Pincus and Singer [23]. However, it has two obvious limitations. First, because there are no self-matches, C-ApEn is not always defined. Second, there is “direction dependence” of C-ApEn analysis due to its template-wise approach. Then, Richman and Moorman [24] proposed cross sample entropy (C-SampleEn) between two time series. C-SampleEn is not direction dependent since it does not use a template-wise approach when estimating conditional probabilities. However, due to the same reason as C-ApEn that it does not count self-match, the definition of C-SampleEn is not always guaranteed. Recently, Xie et al. [25] proposed a new measure called cross-fuzzy entropy (C-FuzzyEn), which was derived from fuzzy entropy [26] that was based on the concept of fuzzy sets. Contrary to the common practice in C-ApEn and C-SampleEn, the similarity between any two embedded vectors is not measured by a Heaviside function, but with an exponential function. Thus, the discontinuity caused by the hard boundary of the Heaviside function is eliminated. As a result, C-FuzzyEn is always defined, and the choice of the parameters is assigned with more freedom.

The main purpose of this paper was to investigate the utility of the cross-fuzzy entropy for measuring gait symmetry using gait rhythm signals. The gaits of the patients with Parkinson's disease (PD) and the healthy control (CO) subjects were analyzed in this study. Parkinson's disease is a typical neurodegenerative disorder related to the central nervous system, and one of the main symptoms at its early stage is gait disorders, such as reduced stride length [27], freezing of gait [28], and increased gait variability [29]. The gait symmetry problem of PD patients was also investigated in previous studies [30, 31], and it has been reported that the degree of asymmetry of the PD patients' gaits was larger than that of the normal gaits. In the present study, we reinvestigated the quantification of gait symmetry for PD patients and CO subjects. We hypothesized that the degree of symmetry can also be measured by using the novel gait symmetry measure that was based on C-FuzzyEn. With the encouraging results obtained in the experiments, we hope this study can provide a useful method for evaluating the abnormal gait of PD patients, especially at its early stage.

## 2. Methodologies

### 2.1. Gait Dataset

The gait dataset used in this study was contributed by Hausdorff et al. [32]. It includes gait data from fifteen PD subjects aged 44–80 years (age mean ± standard deviation, SD: 66.8 ± 10.9 years; 10 males and 5 females) and sixteen healthy CO subjects aged 20–74 years (age mean ± standard deviation, SD: 39.3 ± 18.5 years; 2 males and 14 females). According to the experimental protocol, the subjects were asked to walk at their normal pace along a straight hallway that was 77 m in length for 300 s. The gait signals were measured with ultrathin force-sensitive switches placed inside each subject's shoes. Seven different gait rhythm signals for left or right limb were calculated with the algorithm proposed in [33]. In the present study, we have interest only in left and right stride interval (time from initial contact of one foot to the immediate subsequent contact) time series. To remove the outliers data points caused by the turnaround at the end of the hallway, a preprocessing method [34] was also applied.

### 2.2. Definition of Cross-Fuzzy Entropy

Since cross-fuzzy entropy is developed on the basis of cross sample entropy, we first introduce the cross sample entropy and then point out how cross-fuzzy entropy differs from cross sample entropy. By this way of establishment, it is believed that the comparison of these two cross entropies can be more impressive.

Given two one-dimensional discrete time series with equal length, {*u*(*i*) : 1 ≤ *i* ≤ *N*} and {*v*(*i*) : 1 ≤ *i* ≤ *N*}, the definition of cross sample entropy is given as follows [24]:

(1) Form the vectors:
  (1) xmi=ui+k:0≤k≤m−1,ymj=vj+k:0≤k≤m−1.
(2) The distance between two such vectors is defined as
  (2) dxmi,ymj=maxui+k−vj+k:0≤k≤m−1.
(3) Define *B*_*i*_^*m*^(*r*)(*v*||*u*) = (*N* − *m*)^−1^∑_*j*=1_^*N*−*m*^ *θ*(*d*[*x*_*m*_(*i*), *y*_*m*_(*j*)], where *θ*(·) is a Heaviside function and is given as
  (3) $$\begin{array}{c} \theta \left(\;d\;\right)=\left\{\begin{array}{cc} 1, & if\;\;d<r\\ 0, & otherwise, \end{array}\right. \end{array}$$
 where *r* is a threshold value.
 Thus, *B*_*i*_^*m*^(*r*)(*v*||*u*) can be deemed as the number of *y*_*m*_(*j*) within *r* of *x*_*m*_(*i*) divided by (*N* − *m*), and the distance threshold *r* controls the similarity of two vectors.
(4) Define *B* ^*m*^(*r*)(*v*||*u*) = (*N* − *m*)^−1^∑_*i*=1_^*N*−*m*^ *B*_*i*_^*m*^(*r*)(*v*||*u*), and similarly *B* ^*m*+1^(*r*)(*v*||*u*) is defined on the vectors *x*_*m*+1_(*i*) and *y*_*m*+1_(*i*). Then, cross sample entropy is given as
  (4) $$\begin{array}{c} C\text{-SampleEn}\left(\;m,r,N\;\right)=-\ln\left\{\;\frac{\left[B^{m}\left(r\right)\left(v||u\right)\right]}{\left[B^{m+1}\left(r\right)\left(v||u\right)\right]}\;\right\}. \end{array}$$

Notably, there are two modifications in the cross-fuzzy entropy (C-FuzzyEn) proposed by Xie et al. [25] to the above C-SampleEn. First, the Heaviside function that measures the similarity of two vectors is replaced by an exponential function:

(5) $$\begin{array}{c} \theta \left(\;d,n,r\;\right)=\exp\left(\;-\frac{{\left(d\right)}^{n}}{r}\;\right), \end{array}$$

where *r* controls the width of the exponential function and *n* determines the gradient of its boundary. Thus, C-FuzzyEn can be written as C-FuzzyEn(*m*, *n*, *r*, *N*) to include its different parameters. We set *n* to be 2 in this study according to the suggestion given in [26]. By this fuzzy function, the discontinuity caused by the hard boundary of the Heaviside function can be eliminated. Second, to highlight the effect of vector's shape instead of the absolute values in the fuzzy-based similarity measurement, the baseline is subtracted from the vector; that is,

(6) $$\begin{array}{c} x_{m}\left(\;i\;\right)=\left\{\;u\left(\;i+k\;\right)-\overset{-}{u}\left(\;i\;\right):0\leq k\leq m-1\;\right\}, \end{array}$$

where the baseline $\overset{-}{u}(i)$ is the average of all *u*(*i* + *k*), 0 ≤ *k* ≤ *m* − 1. Similar preprocessing is performed on *y*_*m*_(*j*), *x*_*m*+1_(*i*), and *y*_*m*+1_(*j*).

### 2.3. Performance Tests of C-FuzzyEn

The performance of C-FuzzyEn was tested on some typical simulation signals, such as i.i.d. uniform random numbers and the MIX(*p*) processes. The MIX(*p*) process [35] is a composite of stochastic and deterministic components. For fixed 0 < *p* < 1 and a sine wave signal with *N* points, the MIX(*p*) signal is formed by substituting the randomly chosen *p* × *N* points of the sine signal with the i.i.d. random numbers. In all the following experiments, to highlight the statistical stability of C-FuzzyEn, the time series pair was generated 200 times randomly for a fixed parameter set, and then the mean and the standard deviation (SD) of the C-FuzzyEn values were computed. As a comparison, C-SampleEn was also calculated with the same testing process.

#### 2.3.1. Effect of Parameter r and *N*

It can be found from the definition that the most relevant parameters of C-FuzzyEn are parameter *r* and data length *N*. The influence of these two parameters on the calculation of C-FuzzyEn was evaluated by two experiments. We first evaluated the effect of parameter *r*. The testing signal pairs were an i.i.d. uniform random time series and a MIX(0.6) time series. The embedded dimension *m* was set to be 2, and the data length *N* was first set to be 100 and then 50. As for parameter *r*, it varied from a predefined value set of [0.01 : 0.01 : 0.1] ∪ [0.11 : 0.1 : 1], where *d*_1_ : *d* : *d*_2_ meant that the value varied from *d*_1_ to *d*_2_ in steps of *d*. The second experiment was designed to evaluate the influence of the data length. Two i.i.d. random uniform numbers of different lengths were examined. Data length *N* ranged from 50 to 500 in steps of 50, parameter *r* was set to be 0.3, and the embedded dimension *m* was set to be 2 and 3 successively.

#### 2.3.2. Relative Consistency Analysis

Let *θ* denote a parameter of the given cross entropy algorithm. Given two pairs of time series, (*X*_1_, *Y*_1_) and (*X*_2_, *Y*_2_), then the relative consistency is defined in the following way: if there is *θ*_0_ ∈ *θ*, inducing the cross entropy of (*X*_1_, *Y*_1_) to be lower than that of (*X*_2_, *Y*_2_), that is, Algorithm_(*X*_1_, *Y*_1_)_(*θ*_0_) < Algorithm_(*X*_2_, *Y*_2_)_(*θ*_0_), then, for all *θ*_*k*_ ∈ *θ*, Algorithm_(*X*_1_, *Y*_1_)_(*θ*_*k*_) < Algorithm_(*X*_2_, *Y*_2_)_(*θ*_*k*_) should be true. We tested the relative consistency of C-FuzzyEn by using the following two pairs of time series: the [MIX(0.2), MIX(0.3)] pair and the [MIX(0.3), MIX(0.4)] pair. Since the MIX(0.4) time series should be more disordered than MIX(0.2) time series, it was hypothesized that the cross entropy of the [MIX(0.2), MIX(0.3)] pair should have a lower value than that of the [MIX(0.3), MIX(0.4)] pair for all the parameters. The data length was set to be 100, 50 in sequential order, while in both cases parameter *r* was changed from the set of [0.01 : 0.01 : 0.1]∪[0.11 : 0.1 : 1].

### 2.4. Gait Symmetry Analysis Using C-FuzzyEn

To apply C-FuzzyEn on the gait stride interval signals for the analysis of gait symmetry, there were three parameters to be set up. The first parameter was the embedded dimension *m*, that is, the length of the comparing vectors. Since the data series used in this study were very short (with a length from 169 to 269), we used *m* = 1 in order to calculate the frequency of *m* and (*m* + 1)-component vectors with sufficient statistical accuracy. Then, the value of parameter *r* was chosen from a predefined value set, that is, [0.0001 : 0.0001 : 0.001]∪[0.002 : 0.001 : 0.01]∪[0.01 : 0.01 : 0.1], based on the gait data precision and the results of some preliminary experiments. During the above setting process of parameter *r*, data length *N* was fixed to be 150 since it was close to the maximum length of some gait sequences used in this study. By parameter selection, we hope to find out a parameter set that can provide the largest separation between the C-FuzzyEn values for the normal gait and that for the PD patients' gait.

### 2.5. Statistical Analysis and Classification of Gait Patterns

SPSS software (Version 17.0, SPSS Inc., Chicago, IL, USA) was adopted for all statistical analyses. The continuous and categorical variables between the groups were compared using Mann-Whitney *U* test. A *p* < 0.05 was considered statistically significant.

To implement the classification of Parkinson gait and normal gait with the proposed gait symmetry feature, the popular support vector machine (SVM) classifier was utilized in this study. The SVM classifier introduced by Vapnik [36] is the first implementation of structural risk minimization (SRM), which is a theory that enforced the selection of the optimal learning model from a subset of models. To set up a linear separating hyperplane for nonlinear problems, SVM employed kernel methods to map data to a higher dimensional feature space. In the present study, the popular radial basis function (RBF) kernel was adopted.

The classification performance was evaluated by the leave-one-out (LOO) cross-validation method. Moreover, the classification results were measured by sensitivity, specificity, and accuracy. The area under the receiver operator curve (ROC), as a well-established index of diagnostic accuracy, was also calculated by using the software ROCKIT provided by the University of Chicago, Chicago, IL, USA [37].

## 3. Results

### 3.1. Results of Performance Tests for C-FuzzyEn

#### 3.1.1. Effect of Parameter *r* and *N*

**Figure 1:**
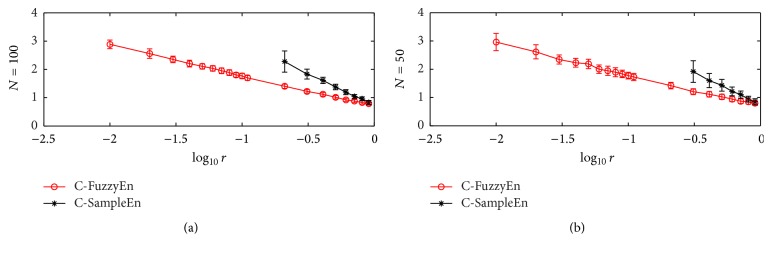
The relationship of C-FuzzyEn(2,2, *r*, *N*) and C-SampleEn(2, *r*, *N*) with parameter *r* for a time series pair composed of a MIX(0.5) signal and a time series of i.i.d. uniform random variables. Circles or stars are the mean value, while bars correspond to the standard deviation for 200 runs of testing. The data length *N* is 100 and 50 for (a) and (b), respectively.

Figure 1 illustrates the calculation results of C-FuzzyEn(2,2, *r*, *N*) and C-SampleEn(2, *r*, *N*) with parameter *r* changing from the value set of [0.01 : 0.01 : 0.1]∪[0.11 : 0.1 : 1]. In either Figure 1(a) (*N* = 100) or Figure 1(b) (*N* = 50), it can be found that C-SampleEn gives no value when *r* falls below a certain value, and the smaller the value of *N* is, the larger the minimum value of *r* is needed to ensure the definition of C-SampleEn. In contrast, such problems do not bother the calculation of C-FuzzyEn, and the choice of parameter *r* and *N* is much more free.

**Figure 2:**
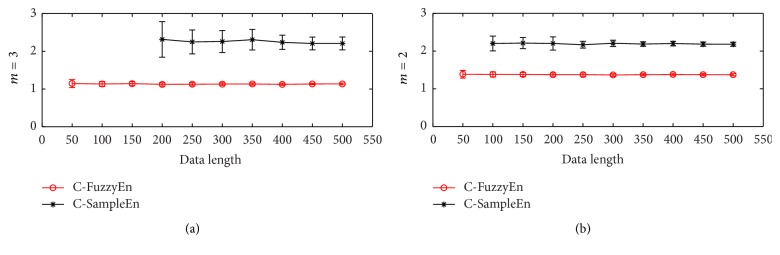
The relationship of C-FuzzyEn(*m*, 2,0.3, *N*) and C-SampleEn(*m*, 0.3, *N*) with data length *N* for two time series of i.i.d. uniform random variables. Circles or stars are the mean value, while bars correspond to the standard deviation for 200 runs of testing. The embedded dimension *m* is 3 and 2 for (a) and (b), respectively.

Figure 2 shows the relationship of C-FuzzyEn and C-SampleEn with data length *N*. The C-FuzzyEn statistics give values for all the data lengths from 50 to 500; however, C-SampleEn fails to work when *N* < 200 for embedded dimension *m* = 3 and *N* < 100 for *m* = 2. In addition, for 200 runs of testing, the standard deviation of the C-FuzzyEn values at each *N* is very small (the mean of the SD value is 0.05 and 0.04 for *m* = 2 and 3, resp.) and the mean values at different data length are practically constant as it is supposed to be like that (the SD value of the mean values is 0.0056 and 0.0068 for *m* = 2 and 3, resp.). In contrast, the values of C-SampleEn display large fluctuations especially when data length *N* is small (the mean of the SD value is 0.10 and 0.27 for *m* = 2 and 3, resp.), and it is manifested more obviously when *N* is smaller. From the above comparison, it is clear that C-FuzzyEn has less dependence on the data length. This property has potential for its usage in gait signals since it is hard to sample a long and continuous gait time series due to the muscle fatigue, especially for those patients with limited walking ability.

#### 3.1.2. Relative Consistency Analysis

**Figure 3:**
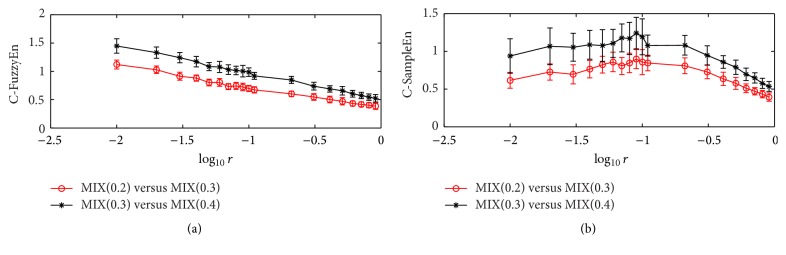
The relative consistency performance of C-FuzzyEn and C-SampleEn on the MIX(0.2) versus MIX(0.3) pair and the MIX(0.3) versus MIX(0.4) pair. Circles or stars are the mean value, while bars correspond to the standard deviation for 200 runs of testing. The data length *N* is 100, and embedded dimension *m* is 2.

**Figure 4:**
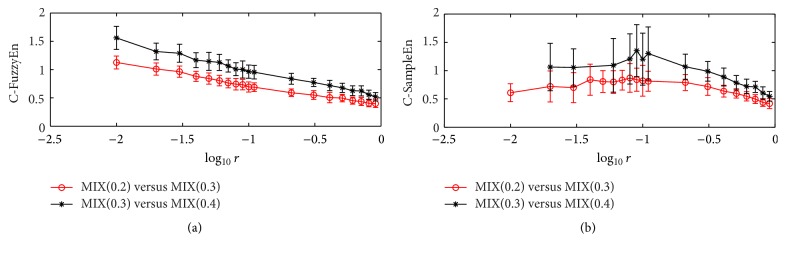
The relative consistency of C-FuzzyEn and C-SampleEn on the MIX(0.2) versus MIX(0.3) pair and the MIX(0.3) versus MIX(0.4) pair. Circles or stars are the mean value, while bars correspond to the standard deviation for 200 runs of testing. The data length *N* is 50, and embedded dimension *m* is 2.

Figures 3 and 4 display the testing results of the relative consistency analysis where the data length is 100 and 50, respectively. It can be clearly seen that the C-FuzzyEn statistics for the [MIX(0.2), MIX(0.3)] pair are consistently lower than that for the [MIX(0.3), MIX(0.4)] pair when *r* takes values from 0.01 to 1. However, the large fluctuations of the values at different *r* value cause the C-SampleEn statistics for the [MIX(0.2), MIX(0.3)] pair to be sometimes larger than that for the [MIX(0.3), MIX(0.4)] pair, though the mean values are always lower for the former than the latter. In addition, by comparing the results in Figures 3 and 4, it can be found that the fluctuation magnitude of C-SampleEn at different *r* value increases significantly when the data length decreases. For instance, the mean of the SD value for the [MIX(0.3), MIX(0.4)] pair is 0.1549 in Figure 3, while it is 0.2772 in Figure 4. Similar to the results in the previous testing, C-SampleEn fails to work when the data length is very short and parameter *r* falls below a certain value, as shown in Figure 4.

### 3.2. Results of C-FuzzyEn on Gait Signals

**Figure 5:**
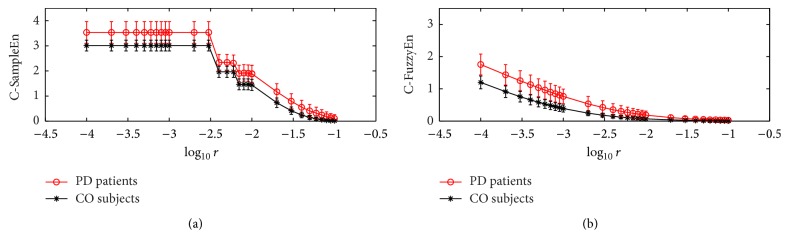
C-FuzzyEn(1,2, *r*, 150) and C-SampleEn(1, *r*, 150) as functions of *r* for left and right stride interval time series. Circles or stars are the mean value, while bars correspond to the standard deviation for each group of gaits.

The results of applying C-FuzzyEn(1,2, *r*, 150) on the left and right stride interval signals are shown in Figure 5, and the results of C-SampleEn(1, *r*, 150) are displayed in the same figure as a comparison. It can be seen that the mean values of both C-FuzzyEn and C-SampleEn for the PD patients are generally higher than that for the normal subjects, while for C-SampleEn the overlapping degree between the PD group and the CO group is greater than the case for C-FuzzyEn. The results, on the one hand, indicate that the PD patients' gait is more asymmetric than the normal gait with respect to the stride interval signal, consistent with the findings in previous studies [13, 31]. On the other hand, the less overlapping between the two groups of gaits demonstrates that C-FuzzyEn has better self-consistency than C-SampleEn when it is applied on two groups of time series pairs with different synchrony or symmetry, especially when the data length is very short [38].

We also observed that in Figure 5 the C-FuzzyEn values for the PD group and the CO group had a tendency to be near zero and equal as parameter *r* increased. This result can be explained as follows: when parameter *r* takes a large value, the width of the membership function will broaden quickly; thus, the membership values will approach one for most distance values. Consequently, the numerator and denominator are both close to one, which leads to very small values of C-FuzzyEn. Therefore, to obtain the largest separation of the two groups, parameter *r* should not take a very large value. We then applied the Mann-Whitney *U* test on the C-FuzzyEn values between the two gait groups by taking the value of parameter *r* from the set [0.0001 : 0.0001 : 0.001]∪[0.002 : 0.001 : 0.01]. It was found that the minimum *p* value of 1.27 × 10^−6^ was obtained when parameter *r* was set to be 0.004. Therefore, we took the value of parameter *r* to be 0.004 as it provided the largest separation between the two groups from the perspective of statistics.

**Figure 6:**
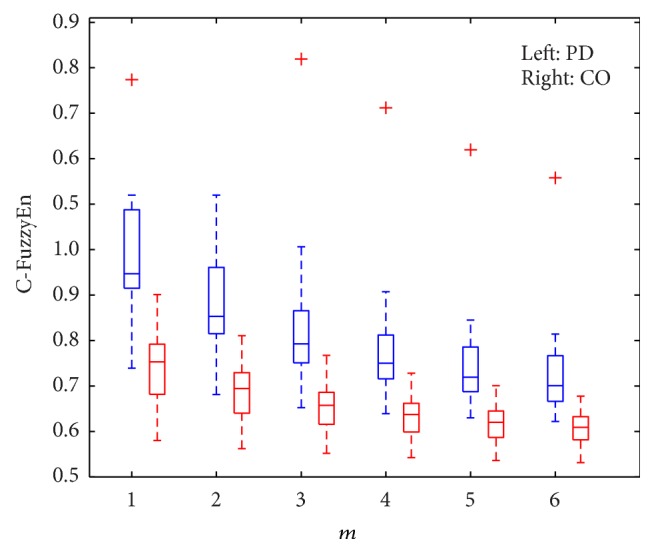
The relationship of C-FuzzyEn(1,2, 0.004,150) with parameter *m*.

For gait stride interval signals, the relationship of C-FuzzyEn with parameter *m* was also investigated in the experiments. The results are shown in Figure 6. It can be seen that the values of C-FuzzyEn have a tendency to decrease for both groups of gait signals as the value of *m* increases. This result is not surprising. This is because when *m* is increased, that is, at a more coarse time resolution, the length of the vector becomes longer correspondingly. Thus, the possibility of several adjacent vectors with the same similarity is increased as they have more overlapping parts. The increase of similar vectors and the decrease of the total vectors together lead to the reduction of the C-FuzzyEn value.

### 3.3. Results of Classification Experiments

**Figure 7:**
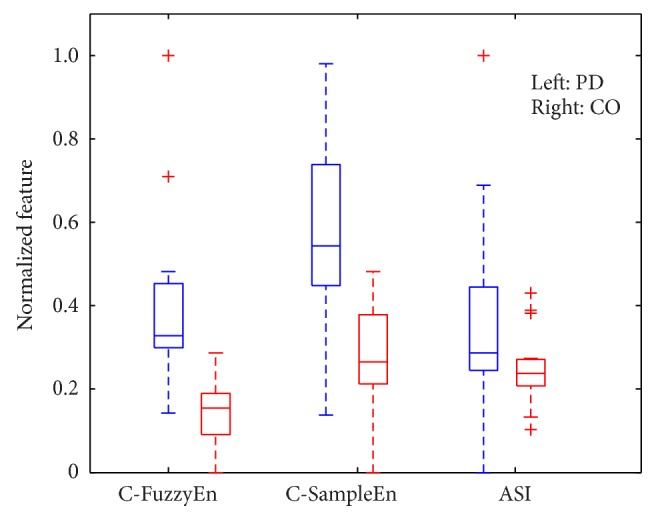
Boxplots of C-FuzzyEn(1,2, 0.004,150), C-SampleEn(1,0.004,150), and ASI feature calculated from the left and right stride interval time series pairs of the PD patients and the CO subjects.

Figure 7 shows the boxplot of the C-FuzzyEn(1,2, 0.004,150) values for the left and right stride interval time series pairs, which are associated with the PD patients and the healthy CO subjects. For a comparison, the boxplots for the other two features, that is, C-SampleEn(1,0.004,150) and ASI feature, are also illustrated in Figure 7. For each feature, all the values are normalized to a range between 0 and 1 by using a min-max normalization method [39]. It can be observed that the C-FuzzyEn values for the normal gaits are congregated in a small range (from 0.09 to 0.19), while for the PD patients' gaits the values are dispersed more widely. Similar distributions also exist for the other two features. Such results indicate that, for the PD patients with different severity levels, significant differences also exist for the degree of gait asymmetry. From Figure 7, one can also find that there is obvious overlapping between the PD group and the CO group for the ASI feature, which indicates that ASI feature is not a good classification feature in the present study. As for C-SampleEn feature, the overlapping is not so obvious, but one can still find that the box of the PD group is closer to the box of the CO group compared with the case for the C-FuzzyEn feature.

Table 1 presents the classification results of C-FuzzyEn(1,2, 0.004,150), C-SampleEn(1,0.004,150), and ASI feature. As shown in Table 1, the accuracies provided by the two cross entropy-based features, that is, C-FuzzyEn and C-SampleEn, are relatively higher than that by the ASI feature. Such results indicate the suitability of cross entropy in expressing the gait symmetry. The best classification performance is provided by C-FuzzyEn(1,2, 0.004,150) with overall accuracy, sensitivity, and specificity of 96.77%, 93.33%, and 100%, respectively. The corresponding ROC area was also calculated, which was 0.968 with a standard error of 0.041. This encouraging classification performance implies that the gait symmetry measure based on C-FuzzyEn may be positively considered as an indicator for the analysis of PD gait patterns.

In the classification experiments, we also investigated how embedded dimension *m* and data length *N* influenced the classification accuracy. As shown in Figure 6, the C-FuzzyEn values for the PD group and that for the CO group also have a tendency to be closer as *m* increases, which is due to the statistical inaccuracy caused by large *m* when the data length is small. Thus, the discrimination of different groups becomes more subtle. Our experimental results showed that when *m* was equal to 6, the best classification accuracy we obtained was 80.64%. To investigate the effect of data length *N* on the values of C-FuzzyEn(1,2, 0.004, *N*), we changed *N* from 10 to 160 with a step of 10 since the minimum data length in our gait dataset was 169. The experimental results showed that significant difference of C-FuzzyEn values existed all the time for all data lengths in the above range; however, when *N* was smaller than 40, the overlapping of the two groups of C-FuzzyEn values increased considerably, thus leading to a relatively poor accuracy of less than 80%.

## 4. Discussion

As a generalization of entropy, the cross entropy represents the synchronicity of patterns embedded in two time series. If two time series have more similar patterns, then the cross entropy should have a lower value. Otherwise, a higher value indicates that the pattern structures between them have a huge difference. As an example, the cross entropy of the [MIX(0.2), MIX(0.3)] pair should be lower than that for the [MIX(0.3), MIX(0.4)] pair by definition. However, the calculation of most entropies often requires a long time series to approximate the theoretical value, which makes it difficult to be applied to practical time series often with short length. Different efforts [23, 24] have been devoted to designing an entropy that has less dependence on the data length. The cross-fuzzy entropy (C-FuzzyEn) is a recently proposed one that is based on fuzzy set theory and has similar definition to the cross sample entropy (C-SampleEn). It obtains better performance by replacing the Heaviside function of C-SampleEn with the exponential function when measuring the similarity of two vectors with equal length. By this way, the problem caused by the rigid boundary of Heaviside function is eliminated, leading to more relative consistency and more freedom of parameter selection as compared to C-SampleEn.

Though cross entropy does not describe time synchronicity of two signals, its application to the analysis of gait symmetry is reasonably effective. As we know, a normal gait is walking with a periodic, symmetric pattern between the left and right limb; hence, the measured kinetic or kinematic bilateral signals should have a synchronous and periodic structure [40]. It means that there are many common patterns between the pair of signals; thus, the cross entropy of the left and right gait signals for normal walking should be lower than that for an abnormal gait. This hypothesis was verified by applying the C-FuzzyEn measure to analyze the gait symmetry problem using the left and right stride interval time series. A gait dataset collected from 15 PD patients and 16 CO subjects was considered in the present study. The statistical analysis demonstrated that the C-FuzzyEn values for the PD patients' gait were significantly higher than those for the CO subjects, which were consistent with previous findings that the gait of PD patients is more asymmetric than that of the CO subjects.

The classification accuracy obtained in this study for differentiating PD gait from normal gait is also comparable to those reported in other studies [29, 41] on the same gait dataset. Daliri [41] used a genetic algorithm to select the best feature subset for classification from a total of 28 features extracted from gait rhythm signals. The best feature set for classification of PD gaits and normal gaits included the left swing interval, left stance interval, and double support interval, and the results of the specificity, sensitivity, and accuracy obtained by the SVM classifier are 89.76%, 89.79%, and 89.33%, respectively. In another similar study, Wu and Krishnan [29] calculated the standard deviation (*σ*) of the stride interval time series based on the probability density function obtained with the nonparametric Parzen-window method, and the signal turns count (STC) of the same time series was also computed. By using SVM classifier and the feature vector formed by STC and *σ*, the classification accuracy and ROC area they reported were 90.32% and 0.952, respectively. From the above comparisons, one can find that with an overall accuracy of 96.77% the proposed gait symmetry measure based on cross-fuzzy entropy is a very promising feature used for classifying PD gait and normal gait.

Some limitations of this study should be acknowledged. First, the size of the current database is small, which limits the test of the generalization ability of the SVM classifier [11]. To better evaluate the classification performance of the proposed feature, more samples need to be added to form a training dataset and a separate testing set in the future studies. Second, in the current database, since the subgroups with different age ranges, sexes, and severity levels have relatively few subjects, it is hard to test the performance of the proposed gait symmetry measure in differentiating those subgroups. To address this problem, future studies need to balance the number of subjects in each subgroup when recruiting more subjects.

## 5. Conclusion

This study investigated the usage of cross-fuzzy entropy for analyzing gait symmetry/synchrony problem. By testing on several simulation signals, we demonstrated that a better performance was obtained by cross-fuzzy entropy as compared with cross sample entropy. On a gait dataset with 15 PD patients and 16 normal subjects, we verified that significant difference (with a minimum *p* value of 1.27 × 10^−6^) existed between such two groups for the gait symmetry measured by C-FuzzyEn values, which were calculated from the left and right stride interval signals. We also realized SVM classifier by using the proposed gait symmetry feature; the classification results evaluated by leave-one-out cross-validation method showed accuracy of 96.77%, sensitivity of 93.33%, specificity of 100%, and ROC area of 0.968.

## Figures and Tables

**Table 1:** Summary of the classification results for PD patients and CO subjects using C-FuzzyEn(1,2, 0.004,150), C-SampleEn(1,0.004,150), and ASI feature calculated from the left and right stride interval time series. The results were obtained by using RBF-kernel-based SVM classifier and the LOO cross-validation method. TP, TN, FP, and FN denote true positives, true negatives, false positives, and false negatives, respectively.

Feature	Positive class (PD)		Negative class (CO)		Specificity (*S*_*p*_)	Sensitivity (*S*_*n*_)	Accuracy (*C*_*a*_)
	TP	FN	TN	FP			
C-FuzzyEn	14	1	16	0	100%	93.33%	96.77%
C-SampleEn	13	2	15	1	93.75%	86.67%	90.32%
ASI	11	4	13	3	81.25%	73.33%	77.42%
